# New Researches with Acetanilide

**Published:** 1888-04

**Authors:** 


New Researches with Acetanilide.—Acetanilide (antifebrin) has been recently subjected to a careful experimental and clinical investigation by Herczel, of Heidelberg. In rabbits, the drug produced grave toxic symptoms only when given subcutaneously and in larger doses. Animals, when given larger quantities of antifebrin for some time continuously, presented symptoms of fatty degeneration of the heart, liver and kidneys. The blood, particularly, becomes altered by the drug; there is a decrease of the hæmoglobin and the alkalescence of the blood. The spinal cord is depressed by the influence of the drug. Antifebrin has become the routine anodyne at the Heidelberg clinics, and is exhibited when all other remedies have failed. It has been found particularly useful in neuralgia of the face, the ears, the muscles, and in intercostal neuralgia. In the radiating pains of ostitis, periostitis, carious processes, vesical polypi, and in hemicrania, the drug has also yielded gratifying results. Its somniferous properties, of course, enhance its value. In anæmic persons, the drug is contra-indicated. The daily dose of antifebrin ought not to exceed thirty to thirty-seven grains, and its prolonged administration is to be avoided, to prevent the depraved condition of the blood following upon aniline intoxication.—*Schmidt's Jahrbücher.*

